# Advances in Intraoperative Glioma Tissue Sampling and Infiltration Assessment

**DOI:** 10.3390/brainsci13121637

**Published:** 2023-11-25

**Authors:** Nadeem N. Al-Adli, Jacob S. Young, Katie Scotford, Youssef E. Sibih, Jessica Payne, Mitchel S. Berger

**Affiliations:** 1 Department of Neurosurgery, University of California San Francisco, San Francisco, CA 94131, USA; nadeem.al-adli@ucsf.edu (N.N.A.-A.); jacob.young@ucsf.edu (J.S.Y.); katie.scotford@ucsf.edu (K.S.); jpayne@s.icom.edu (J.P.); 2 School of Medicine, Texas Christian University, Fort Worth, TX 76109, USA; 3 School of Medicine, University of California San Francisco, San Francisco, CA 94131, USA; youssef.sibih@ucsf.edu

**Keywords:** intraoperative margins, intraoperative imaging, tumor margins, glioma, extent of resection, advanced tissue sampling

## Abstract

Gliomas are infiltrative brain tumors that often involve functional tissue. While maximal safe resection is critical for maximizing survival, this is challenged by the difficult intraoperative discrimination between tumor-infiltrated and normal structures. Surgical expertise is essential for identifying safe margins, and while the intraoperative pathological review of frozen tissue is possible, this is a time-consuming task. Advances in intraoperative stimulation mapping have aided surgeons in identifying functional structures and, as such, has become the gold standard for this purpose. However, intraoperative margin assessment lacks a similar consensus. Nonetheless, recent advances in intraoperative imaging techniques and tissue examination methods have demonstrated promise for the accurate and efficient assessment of tumor infiltration and margin delineation within the operating room, respectively. In this review, we describe these innovative technologies that neurosurgeons should be aware of.

## 1. Introduction

Gliomas are infiltrative primary brain tumors for which the standard of care is maximal safe resection followed by concurrent and adjuvant chemoradiation [1,2]. Maximizing the extent of resection is of utmost importance for survival; however, as recent studies have demonstrated, minimizing the neurological deficits is prognostically important as well [3,4]. Surgical expertise in resecting these tumors, which are sometimes nearly indistinguishable from the adjacent normal tissue, is necessary for minimizing post-operative morbidity [5]. Moreover, it is well known that intraoperative adjuncts such as stimulation mapping reduce the risk of injury to critical cortical and subcortical structures [6]. However, at the periphery of resection, identifying the tumor boundaries remains a challenge, and stopping prematurely may result in suboptimal resections, while overly aggressive resections may cause unnecessary postoperative deficits. Therefore, understanding the delineation between tumor and normal tissues (i.e., margins) is essential for the purpose of maximizing resections and, when combined with methods for assessing the functional capacity of surrounding structures, ultimately optimizes the outcomes for glioma patients.

A neuropathologist review of frozen sections can be conducted for the histological evaluation of tumor margins; however, this is an inefficient use of intraoperative time [7]. More advanced techniques involving intraoperative neuroimaging and nuanced tissue examination may offer similar results, but with a minimal processing time and overhead and, potentially, more granular insights into the tissue at the cellular level. Intraoperative neuroimaging has largely focused on the use of magnetic resonance imaging (iMRI), ultrasound (iUS), and fluorescence for maximizing the extent of resection [8,9]. Alternatively, direct tissue examination has been explored using methods, such as stimulated Raman histology (SRH) and mass spectroscopy (MS) [10]. As these tools become more accurate and feasible for intraoperative use, it is important for neurosurgeons to be aware of those that are potentially applicable to their practice. In this review, we describe the advanced imaging and novel tissue examination techniques for the intraoperative assessment of glioma resection margins (Table 1).

## 2. Neoplastic Tissue Identification

### 2.1. Raman Histology

**Figure 1:**
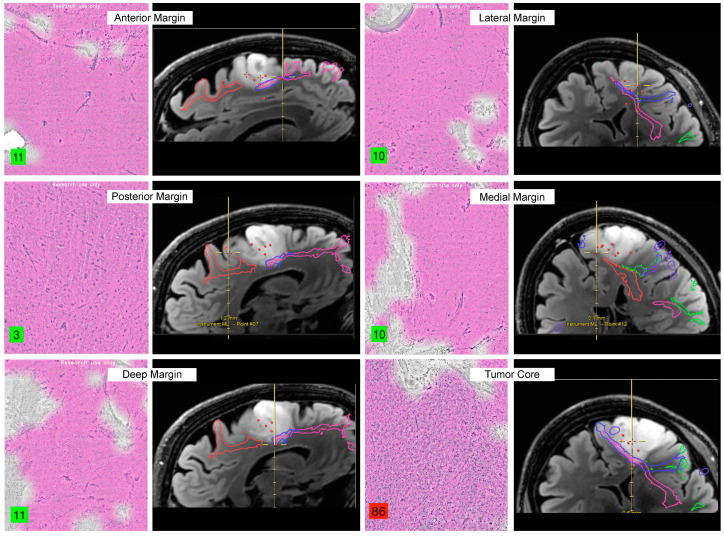
Intraoperative SRH samples obtained for margin assessment with associated MRI localizations. SRH scores (bottom left of each sample), demonstrating strong correlation with sample location, where the highest value was obtained from the tumor core. The different colored lines represent white matter tracts from tractography imaging and area unrelated to SRH.

Stimulated Raman histology (SRH) utilizes the principles of stimulated Raman scattering (SRS) to provide the enhanced molecular imaging of biological tissues. SRH is a label-free, non-destructive technique to gain intraoperative microscopic visualization that can be performed in under three minutes. SRH involves the interaction of two laser beams with the tissue sample. The first laser, known as the pump beam, is tuned to a specific molecular vibration frequency of interest [11]. When the pump beam interacts with the tissue, it promotes the molecules to higher energy states, causing them to undergo SRS [11]. This process generates a coherent anti-Stokes Raman scattering (CARS) signal at a frequency different from the pump beam. The second laser, called the Stokes beam, serves as a reference beam and is slightly detuned from the pump beam [11]. The interaction of the Stokes beam with the excited molecules results in the generation of a CARS signal that is coherent with the pump beam [11]. By detecting and analyzing the CARS signal, SRH provides high-resolution molecular imaging, enabling the visualization of specific biomolecules and cellular structures within the tissues. SRS converts the molecular vibrational properties into histopathologic images by identifying the Raman shifts in the 2800–3100 cm^−1^ range. SRH images use an SRS microscope with a fiberoptic laser to interrogate molecular vibrations from the interacting CH2 and CH3 bonds in the tissue to form histopathologic images that are virtually stained to resemble traditional H&E results instead of spectral data (Figure 1). It is currently one of the only in vivo technologies that can give insights into tumor infiltration are the cellular level and has shown promising results when compared to the gold-standard techniques.

In a recent non-inferiority study of CNS lesions on 18 patients, SRH demonstrated a diagnostic accuracy of 78% compared to 94% for the frozen sections, while the quality of the tissue was equivocal between the two techniques [12]. A larger German study with 73 CNS lesions reported diagnostic accuracies for SRH and hematoxylin and eosin (H&E) of 87.7% and 88.9%, respectively [13]. A prospective study of 82 patients with a CNS lesion, 21 of whom were diagnosed with a glioma, reported no difference in time to diagnosis or diagnostic accuracy when compared to those of the gold standard of IHC [14]. The largest clinical validation study to date included 47 patients and demonstrated a faster time to diagnosis and strong diagnostic concordance with conventional H&E [15]. There was no statistically significant difference between the diagnostic accuracies of SRH versus conventional histopathologic analysis, and the time to diagnosis had a ten-fold deduction with SRH [15]. Considering these promising results, additional groups have started to explore SRH using a handheld visible resonance Raman (VRR) spectroscopy analyzer. In comparison with the conventional methods, the VRR handheld instrument combined with support vector machine learning demonstrated 80% accuracy for binary classifications, with superior sensitivities for differentiating grade 2 gliomas from normal tissues [16]. Nonetheless, additional large, multicentric studies are needed before the widespread intraoperative implementation of SRH.

### 2.2. Fluorescence

In the last twenty years, the implementation of fluorescence-guided surgery (FGS) for the resection of gliomas in eloquent areas has allowed the real-time identification of malignant tissue. Studies have continuously demonstrated the potential for optimizing gross total resection (GTR) with the guidance of three FDA-approved agents: 5-aminolevulinic acid (5-ALA), sodium fluorescein (FNa), and indocyanine green (ICG), to extend the overall survival of glioma patients. The fundamental mechanism of FGS involves detecting the emitted light from fluorescent molecules, known as a fluorophores, which concentrate within the tumor. The advantages, specific properties, and limitations of the three fluorophore agents are discussed below.

#### 2.2.1. 5-Aminolevulinic Acid

5-aminolevulinic acid (5-ALA) is a natural non-fluorescent prodrug of heme synthesis that is subsequently converted into the fluorescent protoporphyrin IX (PpIX), a precursor to heme, and was approved for use in glioma surgery by the United States FDA in June 2017 [17]. Prior to surgery, 5-ALA is administered orally (dose 20 mg/kg) to patients, where it undergoes conversion to PpIX and subsequently accumulates in the malignant glial tissue, given its capability of passing the blood–brain barrier. PpIX exhibits profound light absorption in the violet spectral range (380–420 nm) and emits fluorescence in the red spectral range (620–710 nm) [18]. Intraoperatively, PpIX’s fluorescence can be visualized directly and with an operating microscope fitted with a violet-blue light filter. The core regions of gliomas can display a vibrant red fluorescence, while the surrounding margins exhibit a pink fluorescence, indicating the presence of infiltration. Fluorescence specificity has been shown to be correlated with the glioma tissue density and histological grade [19]. In a study focusing on the fluorescent patterns of 900 patients, 95.4% of the grade IV gliomas demonstrated positivity, while the grade I and II gliomas had positive fluorescence in 26.3% and 24.1%, respectively [20]. Thus, 5-ALA is clearly useful in the setting of high-grade gliomas (HGG), whereas lower-grade gliomas (LGG) may remain poorly differentiated from the normal tissue. In a randomized controlled multicenter phase III trial, Stummer et al. demonstrated an increased EOR and progression-free survival (PFS) rate in HGG patients following FGS with 5-ALA compared to those of the patients who underwent conventional resection with white light [21]. In addition to its limited utility for LGG resections, 5-ALA’s sensitivity and specificity may be limited as well. Recent studies have reported the 5-ALA labeling of non-neoplastic cells within the tumor microenvironment [22] and sensitivities as low as 16% [23]. Additionally, the financial cost of this surgical adjunct can be upwards of USD 12,000 to USD 13,000 [24].

#### 2.2.2. Fluorescein

FNa use in the setting of brain tumor localization has a history dating to 1948, when Moore et al. examined intracranial tumor presence to assist with guiding resections through the direct visualization of fluorophores in a cohort of patients. Sodium fluorescein is a yellow xanthine compound that collects at the site of the malignant tissue, where the blood–brain barrier (BBB) is disrupted [25]. However, the mechanism of its extravasation is not specific to alternative methods, leading to BBB disruption (e.g., injury, edema, etc.) [26]. Fluorescein emits an intense yellow color at the site of blood–brain barrier disruption affected by intracranial tumors. This is a crucial differentiation from 5-ALA, as fluorescein does not integrate with malignant cells [27]. Sodium fluorescein is typically administered at a dose of 5 mg/kg to be visible with an operating microscope fitted with a yellow 560 nm filter or via direct visualization [25]. Due to its dependency on BBB breakdown, sodium fluorescein may not be directly displayed in intracranial tumor types that do not interfere with the BBB, thus limiting its specificity for widespread use [28]. Nonetheless, compared to resections performed under white light, FNa was significantly associated with more complete resections, decreased residual tumor volume, and longer overall and progression-free survival rates [29]. A multi-center, prospective phase II study provided data supporting the safe and effective use of FNa for HGG resection and reported a sensitivity and specificity of 80.8% and 79.1%, respectively [30]. Finally, a recent meta-analysis reported that FNa-guided HGG resection was associated with similar rates of GTR compared to those of 5-ALA and improved rates compared to those of non-fluorescence guided surgery [31].

#### 2.2.3. Indocyanine Green

ICG is a near-infrared (NIR) fluorescent cyanine dye that emits light in the near-infrared region (700–900 nm) of the electromagnetic spectrum. ICG’s mechanism involves binding to plasma proteins with a strong affinity, resulting in effective localization within the intravascular space [18]. Recently, ICG has been used in the setting of glioma resection in a novel technique known as Second Window ICG (SWIG). SWIG involves administering high-dose ICG (5.0 mg/kg) 24 h prior to imaging and resection [32]. Though the mechanism of SWIG is not clear, it has been hypothesized that the administration of ICG 24 h before surgery results in the enhanced accumulation of the dye in the tumor-infiltrated tissue due to increased permeability and retention [33]. The intraoperative visualization of ICG requires the use of a near-infrared surgical detection device. Compared to 5-ALA and FNa, SWIG allows increased tissue penetration and expanded visualization of the tumor through the dura [17]. The SWIG technique has displayed increased stability of the fluorescence signal, which can optimize the consistency of fluorescent imaging in the setting of gliomas. SWIG has a drawback related to the low intensity of the dye’s signal, which ultimately requires the use of a near-infrared (NIR) imaging system with longer exposure times [32].

#### 2.2.4. Future Directions—Targeted Agents

As highlighted in the advantages and limitations for each of the three FDA-approved fluorophores, the optimal fluorescent agent exhibits strong selectivity for the malignant tissue, a low risk of adverse reactions, little to no contraindications, adequate delivery across the blood–brain barrier, and the ability to differentiate between the normal tissue and tumor. Currently, several fluorophores are undergoing clinical trials aiming to cover the aspects that diversify differing tumor subtypes [34]. Novel fluorophores, such as LUM015 [35] Pantimumab-IRDye800CW [36,37], BLZ-100 [36,38,39,40], Cetuximab-IRDye800CW [41,42,43], ^68^Ga-BBN-IRDye800CW [44], ABY-029 [45], and Demeclocycline [46,47], attempt to overcome the limitations associated with the passive accumulation of existing agents by targeting tumor-specific enzymes and ligands to achieve high selectivity for the malignant glial tissue and subsequently improved intraoperative visualization [48,49]. For example, a phase I trial using ^68^Ga-BBN-IRDye800CW, targeting a gastrin-releasing peptide receptor, as a fluorescent probe reported a sensitivity and specificity of 93.9% and 100%, respectively [50]. Similarly, a phase I/II trial evaluating the EGFR inhibitor cetuximab conjugated with IRDye800 reported a sensitivity and specificity of 98.2% and 69.8%, respectively [43].

Additional agents have also demonstrated promise for potential intraoperative use in pre-clinical studies as well. For example, conjugated folic acid-DOTA-ICG has been used to target the folate receptor-α and visualized in real time during the resection of orthotopic GBM models with high sensitivity and specificity [51]. Likewise, compared to the controls, the conjugated chlorotoxin-polymalic acid-ICG produced superior resection margins using NIR-guided intraoperative imaging [52]. Aside from the ICG-based agents, a novel cancer-selective alkyl phosphocholine agent, CLR1502, has demonstrated a superior tumor-to-brain fluorescence ratio compared to that of 5-ALA, highlighting the potential for superior resections with similar targeted agents [53].

### 2.3. Mass Spectrometry

Mass spectrometry (MS) quantifies proteins, lipids, and other metabolites through the measurement of their mass-to-charge ratio with high accuracy and characterizes their spectra at a high resolution [54]. MS-based techniques take advantage of the proteomic changes that precede histological alterations and may provide earlier and more granular differentiation between the tissue types [55,56]. Combined with visualization techniques, MS imaging (MSI) capitalizes on the advantages of MS-based ‘omics’, while adding a spatial component [57,58], which highlights their utility for intraoperative use. A fundamental step involved in MSI is the ionization and desorption of the molecules into charged ions to capture their mass-to-charge ratios and subsequent characteristic spectra. While a comprehensive discussion of all the ionization techniques is beyond the scope of this review, a few have gained popularity for their use in of margin evaluation.

Desorption electrospray ionization mass spectrometry (DESI-MS) for glioma tissue classification was first described by Eberlin et al., who demonstrated the discriminatory ability of DESI-MS lipidomics to not only differentiate between grey matter, white matter, and tumor-infiltrated tissue, but also between the WHO grades as well [59]. More recently, Pirro et al. practically evaluated the implementation of an intraoperative mass spectrometer, where the specimens were analyzed within 3 min of biopsy, and the results were provided prior to standard pathological evaluation [60]. Interestingly, they also identified IDH mutant tumors through the evaluation of the oncometabolite, 2-HG, which represents a potential paradigm shift in the surgical management of these tumors, where more aggressive resections are more favorable [60].

Matrix-assisted laser desorption/ionization MS (MALDI-MS) is an alternative technique that relies on the application of a U-desorbing matrix on the specimen, followed by ionization with a UV laser and subsequent time-of-flight spectrometer measurement of the omic profiles [61]. While this method offers a particularly high spatial resolution, it can be more time-consuming. Despite this limitation, improvements in techniques have reduced the processing time for IDH mutant classification to less than five minutes in some studies [62]. In addition to the IDH status, numerous metabolites and their distributions have been uniquely described using MALDI-based techniques; however, Randall et al. reported a notable distinction in fatty acid metabolism at the edges of tumors versus the core [63,64,65].

Finally, rapid evaporative ionization MS (REIMS) takes advantage of the aerosolization of the tumor tissue in surgical electrocautery and has recently been combined with diathermy instruments, leading to the creation of the iKnife. The rapid mass spectrometric analysis of the gas particles demonstrated 100% diagnostic accuracy during initial testing [66]. In a more recent study, the iKnife was able to distinguish between incremental glioma grades and, importantly, the normal tissue from glioblastoma with 99.3% sensitivity and 100% specificity [10].

## 3. Image-Guided Resection Offers Macroscopic Discrimination

### 3.1. Intraoperative Magnetic Resonance Imaging

Intraoperative magnetic resonance imaging (iMRI) has been utilized in neurosurgery since 1993 to improve navigation and the extent of resection (EOR). The majority of iMRI scanners have the ability to generate T1, T2, FLAIR, and DWI sequences, with some units having DTI capabilities [67]. There have been two randomized controlled trials for the use of iMRI in glioma surgery. In 2011, Senft et al. found a statistically significant increase in the rate of total resections for the iMRI group compared to those of the controls, notably without a difference in the occurrence of postoperative neurological deficits between the two groups [68]. Similarly, in 2014, Wu et al. found a statistically significant increase in the GTR of all the gliomas for the iMRI group compared to that of the control [69]. The EOR was also increased with the use of iMRI in low-grade gliomas, however, to a lesser degree. A multicenter database study found that histopathological specimens acquired after the use of iMRI contained a residual tumor of grade I-IV gliomas in 89–93% of cases [70]. The disadvantages of iMRI include the time and resource requirements. Senft et al. estimated that the use of iMRI increased the length of surgery by one hour. Additionally, the need for substantial operating room space and financial resources render iMRIs unattainable for hospitals with fewer resources.

### 3.2. Intraoperative Ultrasound

Ultrasound was first introduced to neurosurgery in the 1930s and has been used intraoperatively since 1980. The key characteristics of intraoperative ultrasound (iUS) include a lack of radiation exposure, portability, the relative ease of use, and low cost, particularly compared to those of iMRI. iUS provides real-time anatomical guidance without a signficant increase in the operative time. However, the benefits and disadvantages of iUS vary by the type of ultrasound. For example, the application of conventional ultrasound may be limited by the tumor grade. While low-grade gliomas usually have clear borders of hyperechoic tissue with homogeneous internal echoes, HGGs have less clear borders, which limits the value of conventional iUS for higher-grade tumors [8].

#### 3.2.1. Contrast-Enhanced US

Contrast-enhanced ultrasound (CEUS) can improve the application of ultrasound for HGGs due to the differing interactions between the contrast material and types of tissues. Contrast perfusion is relatively quick for HGG, forming a nodular appearance that can help distinguish between the residual tumor and peritumoral edema. A parallel implication is the idea that the time to peak contrast may indicate the tumor grade [8]. A prospective study of 50 brain tumor patients compared the use of 3D ultrasound with versus without contrast. The authors found that the use of 3D CEUS led to more radical resections compared to those of the non-contrast group, notably without increasing the postoperative neurological deficits [71].

#### 3.2.2. Three-Dimensional US

The retrospective analysis of conventional and 3D ultrasound (3DUS) showed that conventional US is more often used for superficial tumors in non-eloquent areas, and 3DUS is more commonly used for deep tumors in eloquent areas. However, the authors concluded that this is dependent on the surgeon’s preference and is not necessarily a recommendation for practice [72]. A 2013 retrospective analysis of one medical center’s use of 3DUS for brain tumor resections suggested the use of 3DUS in place of iMRI for hospitals with fewer resources [73]. Overall, the lack of literature limits recommendations its widespread implementation.

The commonly cited disadvantages of iUS include a resolution lower than that of the other imaging modalities, difficulty with visualizing small or deep tumors, and the appearance of additional echoes in previously radiated patients. Additionally, the value of iUS can depend on the surgeon’s experience and level of comfort. There have been no randomized controlled trials (RCTs) examining the use of intraoperative ultrasound in brain tumor resection nor any RCTs comparing iUS to iMRI [74,75]. As a result, there is a lack of established guidelines for the application of ultrasound in brain tumor resections.

### 3.3. Fluorescence-Guided Resection

The use of intraoperative fluorescence techniques, particularly with high-grade gliomas is well established, and the properties of the different fluorophore agents can be found above. The appropriate dose of fluorescent material, such as 5-ALA, is administered before anesthesia induction. Fluorescence in the tissue is related to the tumor grade, thus guiding the surgeon to the tissue with a malignant morphology. However, it is possible for a low-grade morphology to fluoresce and for high-grade tumors to lack fluorescence. Jaber et al. found positive correlations between the reliability of fluorescence as an indication of the grade and increased age or tumor volume. The fluorescing tissue is identified by the surgeon and resected when possible. The degree to which tissue exhibits fluorescence exists on a spectrum, and categorization depends on the surgeon’s subjective assessment [76,77].

## 4. Nuanced Tissue Examination

### 4.1. Confocal Microscope

Confocal laser endomicroscopy (CLE) is an FDA-approved imaging technique for intraoperative fluorescence visualization. Unlike SRH, CLE does not require the tissue to be excised, and its results can be interpreted in vivo. CLE involves the placement of a light-emitting probe on the tissue of interest, which recaptures the reflected light on a specific plane [78,79]. In combination with fluorescence, recent studies have demonstrated promising results when compared to those of standard pathological diagnosis [80]. Moreover, compared to wide-field imaging, CLE demonstrated the superior detection of 5-ALA and fluorescein sodium [26].

### 4.2. Third Harmonic Generation Microscopy

Similar to mass spectrometry, third harmonic generation microscopy (THGM) is a label-free method for tissue characterization as it relies on the tissues’ susceptibility to the emitted photons as opposed to an extrinsic dye [81]. Recent studies have demonstrated this tool’s ability to identify and quantify the hallmarks of the glioma-infiltrated tissue: increased cellularity, nuclear pleomorphism, and the rarefication of neuropil [82]. Furthermore, when applying a cut-off value, these techniques can identify the tumoral tissue with 96.6% sensitivity and 95.5% specificity. In a study by Blokker et al., they furthered the work conducted by Zhang et al. by implementing a more time-efficient deep learning model, which processed the harmonic generated images and classified the tissue with an average accuracy of 79%, based on the consensus of three pathologists [83]. Despite it performing worse in terms of statistical accuracy, their experiment likely represented a more realistic testing environment.

## 5. Analytical Methods

### 5.1. Big Data and Collaboration

OpenSRH is currently the only publicly available framework with optical histology for cancer [84]. OpenSRH is designed to facilitate the implementation and utilization of SRH in research and clinical settings. It provides an open-source platform that streamlines the entire SRH workflow, from data acquisition to analysis and visualization. OpenSRH offers a user-friendly interface that enables researchers and practitioners to control the laser parameters easily, adjust the imaging settings, and acquire SRH data from tissue samples [84]. It also incorporates advanced algorithms for image processing and analysis, allowing the extraction of valuable molecular information and the generation of high-resolution histological images [84]. By promoting data accessibility, OpenSRH aims to accelerate the adoption and advancement of SRH as a powerful tool for tissue imaging and pathology, fostering collaboration and innovation for tailored patient care.

### 5.2. Machine Learning

As intraoperative technology advances, the data produced are increasing in size and complexity. Combined with the heterogeneity of gliomas, the standard statistical methods are no longer sufficient for extrapolating clinically relevant relationships for the purpose of discriminating tumors from normal tissue. As such, machine learning methods have become fundamental in the analytical phase of these processes [85]. A recent study evaluating the ability of High-Resolution Magic Angle Spinning Nuclear Magnetic Resonance to identify the biomarkers that discriminate between malignant and normal tissues extracted and analyzed the tissue metabolites in an untargeted manner, resulting in spectra that are too granular for standard analyses and potentially too foreign for manual adjustments. The researchers benchmarked various algorithms and reported a random forest method as the most accurate model in distinguishing the two entities. In addition to reporting a median AUC and AUCPR of 87.1% and 96.1%, respectively, their model identified known biomarkers, such as creatine and 2-HG, as important factors in this distinction, further validating the implementation of these machine learning methods [86]. Similarly, the results have been demonstrated using model-based classifiers of optical coherence tomography [87] and ResNetV50 convolutional neural network classifiers from SRH [88]. These highly accurate and efficient models provide unique classification abilities that may extend the utility of these intraoperative tissue analytic methods. For example, it is well known that recurrence presents glioma surgeons with the challenge of distinguishing true progression from pseudoprogression; however, combining CNN and SRH has demonstrated a diagnostic accuracy of 95.8% [89,90]. Recently, other groups have reported the use of intraoperative nanopore sequencing combined with advances in machine learning algorithms to deliver molecularly subclassified diagnoses within 90 min [91,92]. Sturgeon, a transfer-learned neural network, was tested intraoperatively in 25 operations and correctly reported diagnoses in 72% of all the cases and 80% of HGG cases within 45 min of sequencing [91]. As such, combining advanced tissue sampling and assessment techniques with machine learning methods presents a significant potential for obtaining rapid intraoperative diagnoses and, potentially, more specific classifications to aid in surgical decision making.

## 6. Conclusions

The surgical management of gliomas is complicated by the infiltrative nature of these tumors. While maximal safe resection is an essential part of management, preserving the neurological function, while minimizing the residual tumor cells, particularly at the resection boundaries, requires surgical expertise and a deep understanding of the patient’s anatomy. The current methods used for distinguishing tumor-infiltrated from normal brain tissues are disadvantaged by their inefficiencies; however, recent advances in intraoperative imaging, fluorescent guidance, and label-free tissue examination have demonstrated highly accurate and efficient results. Moreover, coupled with open-source data sharing, advanced machine learning analyses have enabled the evaluation of high-dimensional and granular data for the most accurate classification models.

## Figures and Tables

**Table 1:** Methods for intraoperative margin and resection assessment for use in glioma surgery.

Technique	Use	Method	Feasibility Requirements		
			Expertise/Training	Resources	Time
SRH	Margins	Label-free	High	High	Medium
FGS	Margins/EOR	Fluorescent dye	High	Medium	Low
MSI	Margins	Label-free	High	High	High
iMRI	EOR	Radiology	Medium	High	High
iUS	EOR	Radiology	Low	Medium	Low
CLE	Margins	Label-free	High	High	Medium
THGM	Margins	Label-free	High	High	Medium

## Data Availability

Not applicable.
